# Post-Stroke Sleep-Disordered Breathing—Pathophysiology and Therapy Options

**DOI:** 10.3389/fsurg.2018.00009

**Published:** 2018-02-26

**Authors:** David Stevens, Rodrigo Tomazini Martins, Sutapa Mukherjee, Andrew Vakulin

**Affiliations:** ^1^A Flinders Centre of Research Excellence, College of Medicine and Public Health, Adelaide Institute for Sleep Health, Flinders University, Daw Park, SA, Australia; ^2^Neuroscience Research Australia, Randwick, NSW, Australia; ^3^Sleep Health Service, Respiratory and Sleep Services, Southern Adelaide Local Health Network, Adelaide, SA, Australia; ^4^The NHMRC Centre of Research Excellence, NEUROSLEEP, Woolcock Institute of Medical Research, The University of Sydney, Sydney, NSW, Australia

**Keywords:** stroke, sleep apnea, hypopnea, treatment, phenotyping

## Abstract

Sleep-disordered breathing (SDB), encompassing both obstructive and central sleep apnea, is prevalent in at least 50% of stroke patients. Small studies have shown vast improvements in post-stroke functional recovery outcomes after the treatment of SDB by continuous positive airway pressure. However, compliance to this therapy is very poor in this complex patient group. There are alternative therapy options for SDB that may be more amenable for use in at least some post-stroke patients, including mandibular advancement, supine avoidance, and oxygen therapy. There are few studies, however, that demonstrate efficacy and compliance with these alternative therapies currently. Furthermore, novel SDB-phenotyping approaches may help to provide important clinical information to direct therapy selection in individual patients. Prior to realizing individualized therapy, we need a better understanding of the pathophysiology of SDB in post-stroke patients, including the role of inherent phenotypic traits, as well as the contribution of stroke size and location. This review summarizes the available literature on SDB pathophysiology and treatment in post-stroke patients, identifies gaps in the literature, and sets out areas for further research.

## Introduction

Sleep-disordered breathing (SDB) encompasses a range of respiratory sleep disorders which affects ~20% of the middle-aged population (1, 2). The known risk factors for SDB include obesity, age, and male gender (3). SDB is associated with a range of negative health outcomes including excessive daytime sleepiness leading to neurobehavioral and cognitive dysfunction (4), hypertension (5), and diabetes (6).

Stroke is one of the leading causes of morbidity and mortality. Stroke can occur ischemically, where there is a blockage of a cerebral artery, or hemorrhagically, where the cerebral artery has ruptured. Hemorrhagic strokes only comprise 15–20% of strokes, but account for almost half of stroke deaths. There is a strong link between SDB and stroke risk (7); however, the effectiveness of continuous positive airway pressure (CPAP), the gold-standard treatment of SDB, in reducing stroke risk is not supported. For example, a recent large randomized control trial (RCT) did not support CPAP as a risk reduction strategy for recurrent stroke in patients with moderate to severe OSA, although in highly compliant CPAP users, there may be some protective effect (8). SDB is more severe during the acute stroke phase; however, despite improvement over time, 53% of stroke patients demonstrate at least moderate–severe OSA after 1 month according to a meta-analysis (9). Post-stroke acute SDB, however, has received less attention in the literature, but there is emerging evidence that post-stroke SDB is very common and is associated with adverse effects, including poorer stroke recovery outcomes (10, 11). Emerging evidence, however, suggests that CPAP treatment in post-stroke patients may lead to faster functional recovery and reduction in the length of hospitalization and frequency of re-hospitalization (12–14).

It is important to improve our understanding of the pathophysiology of post-stroke SDB in order to initiate appropriate rehabilitation therapy to facilitate faster recovery in stroke patients. This review will briefly summarize and highlight the growing literature on post-stroke prevalence and the clinical presentation of SDB. Furthermore, with the increased use of neuroimaging and a greater understanding of SDB pathophysiology, recent studies have begun examining whether stroke lesion location and size are associated with SDB presentation. Lastly, we will review the emerging novel SDB-phenotyping approaches that may be clinically useful to better characterize the nature of SDB in post-stroke patients and help guide targeted post-stroke therapy.

## Prevalence and Pathophysiology of SDB Post Stroke

**Figure 1:**
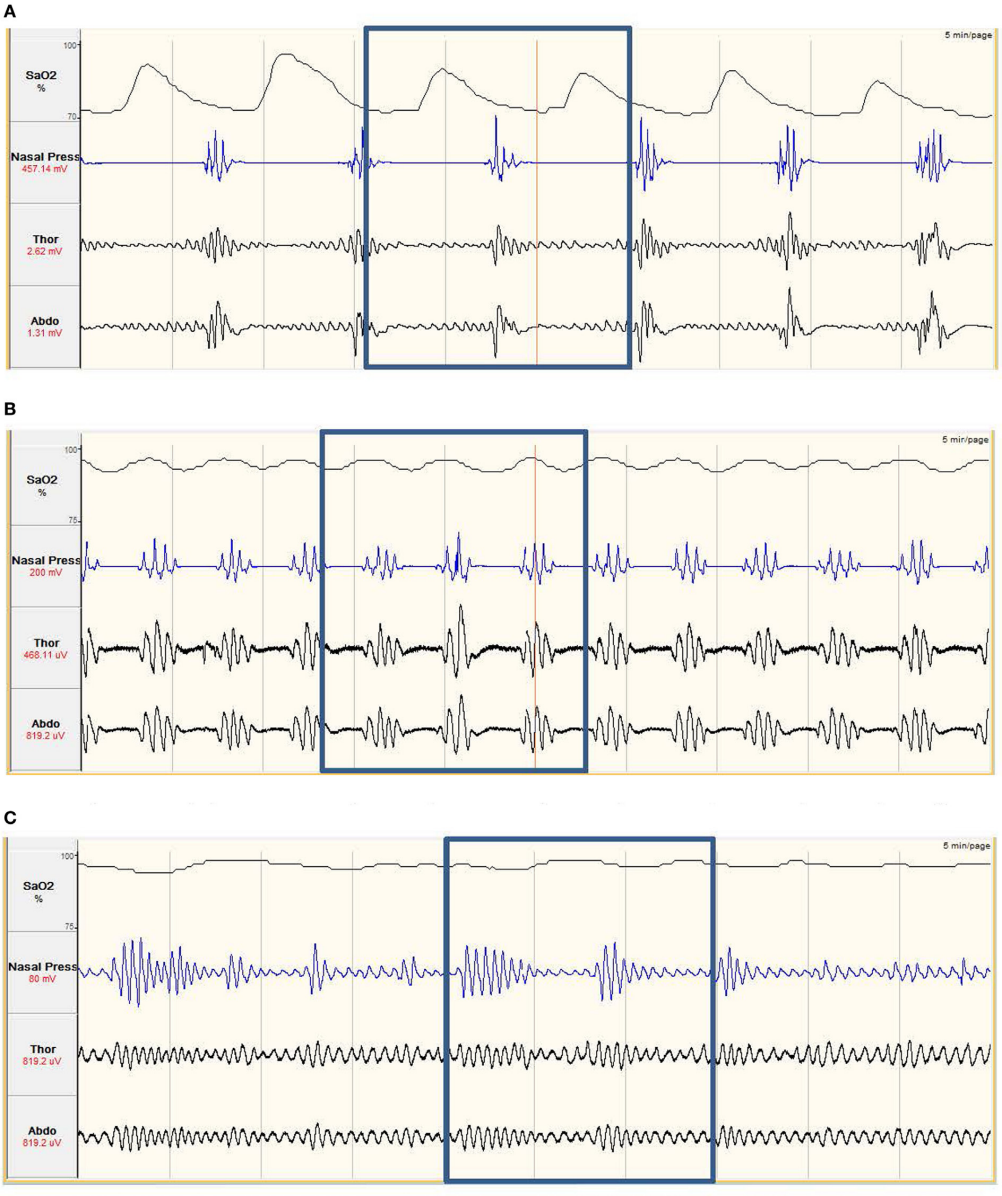
**(A)** Obstructive sleep apnea. The nasal pressure (second tracing from top) goes through periods of ‘flattening’, where airflow has ceased. The abdominal and thoracic respiratory bands (third and fourth tracing) show continued effort to breath, with effort increasing prior to the recovery of breathing. The continued respiratory effort with no airflow implies the airway has collapsed. The oxygen saturation (SaO2%, top tracing) shows periods of desaturation. The desaturation, and recovery, is delayed compared to the respiratory effort. Obstructive apneas occur multiple times in the example. **(B)** Central sleep apnea. The nasal pressure (second tracing from top) goes through periods of ‘flattening’, where airflow has ceased. This is combined with the abdominal and thoracic respiratory bands, which are also ‘flattening’, showing no effort to breathe. This combination of no airflow and no effort to breathe imply the neural drive to breathe is impaired. Central apneas occur multiple times in the example and also result in periods of SaO2% (top tracing) desaturation. **(C)** Hypopnea. The nasal pressure shows periods of increased and decreased breathing, coupled with increased and decreased respiratory effort. As airflow is still maintained but leads to decreases in SaO2% (top tracing) of at least 3%, this is classed as a hypopnea.

The most common form of SDB is obstructive sleep apnea (OSA), which is the reduction (hypopnea) or cessation (apnea) of airflow during sleep as a consequence of upper-airway collapse. Another form of SDB is central sleep apnea (CSA), which is also characterized by cessation of respiration but as a consequence of loss in central respiratory drive and effort, rather than physical upper-airway collapse (Figure 1).

The gold-standard diagnosis for SDB is performed by overnight laboratory polysomnography (PSG), comprising electroencephalography (EEG) and respiratory measures (pulse oximetry, nasal pressure, and respiratory effort). PSG, however, can be cumbersome and time-consuming, making it impractical in some clinical settings such as post-stroke (15). As such, the vast majority of studies examining SDB and stroke use respiratory polygraphy, which is a limited channel sleep study measuring respiratory parameters without EEG. There is good agreement between the limited channel studies compared with gold-standard PSG (16, 17) suggesting that the prevalence estimates for SDB post stroke are reasonable.

### Prevalence of SDB after Stroke Compared to Prevalence in Non-Stroke Patients

There is cross-sectional evidence suggesting that SDB is more common among stroke patients, compared with the general population, with a recent meta-analysis showing that SDB affects >50% of stroke patients (9). Furthermore, studies have demonstrated that stroke patients experience a higher prevalence of CSA (>5 respiratory events/hour) compared with non-stroke SDB patients (18–20).

An important limiting factor in this area is determining whether there was preexisting SDB prior to the stroke and how this may influence post-stroke acute SDB pathophysiology. Few studies have used questionnaires to quantify snoring prevalence prior to the stroke (21, 22), and there are screening questionnaires for SDB that may provide information on whether SDB was present prior to the stroke. Nevertheless, the use of these questionnaires would be subject to recall bias. Ultimately, unless there has been a prior objective clinical diagnosis of SDB, it is very difficult to determine the relationship between prior and post-stroke SDB.

## Treatment of SDB Improves Clinical Recovery in Stroke Patients

Untreated SDB in stroke patients leads to numerous adverse outcomes during acute and chronic recovery from stroke including higher long-term mortality (23–25) and reduced functional outcomes (10, 25, 26).

Traditionally, as the majority of SDB events in the general population are obstructive in nature, this was considered to be caused by a collapsible airway (27, 28). CPAP acts as a pneumatic splint *via* the application of pressurized air to prevent airway collapse. There are different types of positive airway pressure (PAP) machines that can operate at a set pressure, or other machines that can automatically detect the pressure needed to maintain airway patency (known as an auto-CPAP, or APAP). CPAP usage of more than 4 h a night, for at least 70% of nights, is considered clinically compliant (29). Adaptive servo-ventilation (ASV) has in the past been used to treat CSA; however, a recent study has questioned the efficacy of this treatment (30). Furthermore, there has been no research examining the effects of ASV in post-stroke SDB patients.

Successful treatment of SDB by both CPAP (13) and APAP (12) in stroke patients has been shown to improve NIH Stroke Scale. In addition, treatment has been shown to reduce the risk of mortality by 32% (14). Yet, despite the well-documented benefits of PAP, compliance to therapy in general populations remains poor, with several studies showing an average nightly usage of less than 4 h a night, and in many cases, less than half the nights of the week. Furthermore, up to 50% of patients abandon CPAP treatment in the long term (8, 31). Many of the reasons for low compliance in the general population include mask discomfort, anxiety, and difficulty using CPAP therapy (32, 33). Compliance with CPAP appears to be even worse in stroke patients with SDB, with uptake in stroke populations regularly below 50% (23, 34). Furthermore, those that do use CPAP often use it for less than 4 h/night (34–36).

Despite the prevalence of CSA in stroke patients, no study has attempted to treat CSA directly in SDB patients. This is further exacerbated by an RCT highlighting that the previous treatment strategy of CSA, ASV, provided no protection against CSA-related death (30). While CSA is not caused by a collapsed airway, CPAP has been shown to have some efficacy in reducing CSA; however, this is due to addressing non-stroke causes in CSA, such as decreased left-ventricular ejection fraction (37, 38). Thus, it is yet unclear whether CPAP would benefit stroke-related CSA. Nevertheless, given the documented benefits of the treatment of SDB on the functional recovery of stroke patients, it is imperative to develop strategies and care pathways to motivate and encourage SDB treatment in stroke patients. These may include the use of non-CPAP-related treatments in conjunction with novel respiratory phenotyping techniques, which may be more suitable and acceptable for stroke populations during recovery in both hospital and home environments.

### Other Potential Treatments for Post-Stroke SDB

The development of non-CPAP treatments for SDB has been growing. Diet and physical exercise to reduce obesity has shown promise to reduce the severity of SDB in non-stroke populations (39, 40); however, as in general populations, it is difficult to achieve and maintain in non-stroke patients (41, 42). The functional impairment caused by stroke, such as immobility, means weight loss is difficult to implement. Furthermore, during the post-stroke period, stroke patients with variable degrees of hemiparesis often lose muscle mass but increase fat mass (43), leading to obesity, which can worsen SDB.

Obstructive sleep apnea is often more severe in the supine position compared to that in lateral postures (44). Recent technological developments in supine avoidance devices for OSA have shown promising results in terms of efficacy in reducing OSA severity as well as treatment adherence (45, 46). A small RCT involving SDB stroke patients using a supine avoidance pillow showed a significant decrease in apnea/hypopnea index (AHI) while using the pillow; however, larger studies are needed to properly elucidate the effect of supine avoidance on SDB in stroke patients (47). Furthermore, this technique may not be suitable for all stroke patients, particularly those with hemiparesis, as they may not be able to shift their body weight to, or maintain, a lateral position.

Mandibular advancement splints (MAS) move the mandible forward, thereby opening the airway and are effective at reducing OSA in 30–50% of patients (48). This therapy may be beneficial in some stroke patients and may be better suited and tolerated in stroke populations compared to CPAP treatment; however, MAS has not been tested in stroke patients thus far.

Surgical interventions to reduce OSA severity have shown promise in non-stroke populations; however, studies are lacking in this area (49). Furthermore, given the potential complications from stroke, surgery may not be a feasible option.

Oxygen therapy has also been evaluated as a potential treatment option in OSA (50) and would seem to be an appropriate, minimally invasive therapy in post-stroke patients with SDB. The effectiveness of oxygen therapy may be limited, however, with evidence suggesting that it is effective at significantly reducing oxygen desaturation, but may prolong apneic events (50). More recent evidence suggests that only certain OSA patients with particular phenotypic traits respond positively to oxygen therapy (51), highlighting the need for future research on simple phenotyping and therapies in post-stroke patients.

Despite the availability of various treatment options for OSA, there has been very limited research examining the acceptance and efficacy of non-CPAP therapies on improving SDB and functional recovery in stroke patients.

### Phenotyping SDB in Stroke Patients to Inform Novel Treatment Strategies

There are several nonanatomical contributors to SDB, being a low-respiratory arousal threshold, abnormal chemoreflex response to differing carbon dioxide (CO_2_) levels, and low activation of the main upper-airway dilator muscle, the genioglossus (52). This knowledge has allowed for the development of emerging “tailored” treatments for those that have these nonanatomical contributors to SDB. Low arousal threshold can be treated by the use of sedative (53), abnormal chemoreflex has been treated by supplementary oxygen (54), and low activation of the upper-airway dilator muscles can be improved by targeted oral exercises (55). Importantly, these SDB phenotypes can be identified from diagnostic PSG studies (56, 57); however, the characterization of these phenotypes has not been performed in post-stroke SDB patients.

## Does Stroke Lesion Size and Location Impact SDB Presentation?

The three nonanatomical contributors to SDB are controlled through the brainstem. Thus, it is biologically plausible that stroke lesions that involve the brainstem may contribute to SDB. This may explain why stroke patients experience higher rates of SDB (58). If stroke lesion location does contribute to SDB, this knowledge, combined with SDB phenotype information, could potentially be used clinically to inform the optimal mode of therapy for a particular patient.

### Neural Centers of Respiratory Control

Within the brainstem, the medulla oblongata plays the primary role in respiratory control (59). The medulla oblongata contains central chemoreceptors (with peripheral chemoreceptors located in the carotid bodies). The chemoreflex response controls respiration through a loop-gain feedback system depending on the partial pressure of CO_2_ detected (PaCO_2_). In people with “normal” chemosensitivity, an increase in PaCO_2_ will increase respiration, while a decrease in PaCO_2_ will reduce respiration. In those with “abnormal” chemosensitivity, increases in PaCO_2_ lead to above normal increase in ventilation, which can contribute to CSA (60). Lesions to the respiratory centers within the medulla have shown decreased chemosensitivity during wakefulness, sleep, and even exercise (61).^1^

The medulla also receives input from the respiratory center within the pons to innervate the pharyngeal muscles, which play an important role in maintaining the patency of the upper airway, as well as the regulation of diaphragm activity. The largest pharyngeal muscle, the genioglossus, is innervated by the hypoglossal nerve, which originates from the brainstem (62) and acts to pull the tongue forward. SDB patients experience a reduction in genioglossus activation during sleep, which importantly contributes to upper-airway collapsibility (63, 64). Several studies have shown that brainstem strokes affect pharyngeal muscle activity (65, 66) causing dysphagia and can contribute to higher rates and severity of SDB observed in stroke patients. This implies that stroke-induced damage to the respiratory brain regions innervating the genioglossus muscle activity may, at least in part, explain the increase in upper-airway collapsibility in stroke patients.

### Lesion Location

#### Severity and Type of SDB

Early studies examining brainstem strokes showed lesions to this region led to the development of Cheyne-Stokes respiration (periodic breathing occuring whilst awake) (67, 68). Lee et al. was the first to show high levels of CSA in patients who had suffered a brainstem lesion (67). Several case–control studies suggest that there is a link between brainstem stroke and SDB severity and in particular increased CSA severity (69, 70). The limited research in this area, however, means larger prospective studies are needed to make strong conclusion regarding this relationship.

The advent of neuroimaging has allowed for a more detailed examination of the potential role that lesion location might have on SDB in stroke patients. These studies have produced conflicting findings on whether stroke in the brainstem contributes to SDB and whether it contributes to specific types of SDB (obstructive vs central). Infratentorial lesions, which encompass both the brainstem and cerebellum, have been shown to generally result in higher AHI (20, 71) when compared to cortical lesions. Likewise, another study (18) has shown that when compared to cortical strokes, brainstem strokes have been associated with three times greater odds of significant SDB, particularly CSA, along with greater nocturnal desaturations (26). No study has yet examined whether brainstem lesions are associated with changes in respiratory arousal threshold. Given that a low arousal threshold contributes to unstable respiratory control and reduces the likelihood of entering slow-wave sleep, while a high arousal threshold may exacerbate arterial hypoxemia, it is important to determine whether stroke in the brainstem changes arousal threshold.

By contrast, other studies have found that non-brainstem regions might be more related to SDB in stroke patients. For example, Siccoli et al. found that patients who experience total anterior circulatory strokes had the highest AHI compared to other brain regions assessed, with CSA making up 40% of the overall AHI (19). Furthermore, the authors showed that the second highest AHI resulted from strokes in the pons, which is part of the midbrain, where CSA made up only 12% of AHI events. Ahn et al. found that bilateral cerebral lesions were associated with significantly higher SDB severity, while stroke in other regions, including the brainstem, was not associated with SDB, and the authors did not report on the type of SDB (72). De Paolis et al. have hypothesized that cerebral infarctions can lead to increased cranial pressure leading to a reduction in cerebral blood volume, which would precipitate hypocapnia, leading to unstable ventilatory control and CSA (73). This hypothesis may partly explain why another study (74) has showed a spontaneous decrease in SDB, in particular CSA after 3 months, despite not showing SDB severity or the type to be associated with lesion location.

Several studies have also shown that there is no association between lesion location and SDB severity and SDB type (74–78); however, the reasons for this are unclear. It is important to note that brainstem-specific strokes contribute to less than 10% of the number of cortical strokes, limiting the power to demonstrate lesion location impact on SDB (18, 77).

In summary, it appears that the literature on lesion location and SDB is mixed and inconclusive. There is large variation in methodology and patient populations, lesion location definitions, and even the timing of the SDB measurement relative to stroke onset, which may contribute to conflicting results.

#### Brainstem and Pharyngeal Muscles

Despite the importance of the pharyngeal muscles in maintaining upper-airway patency, there has been relatively little research directly examining the potential role of damage to the pharyngeal nerves in post-stroke SDB patients, despite studies showing both brainstem lesions (79) and lesions to the pharyngeal cortex (80), leading to dysphagia. Turkington et al. showed that BMI and neck circumference, but not the presence of dysphagia, contributed to worse SDB severity (81). Brown et al. showed that stroke patients with SDB had a narrower retropalatal distance, which is the size of the opening of the airway, compared to non-SDB. Importantly, dysphagia was not different between SDB and non-SDB stroke patients (82). By contrast, Martinez-Garcia et al. showed that stroke patients with brainstem stroke were 1.73 times more likely to experience dysphagia and a significantly higher number of obstructive apnea events compared to stroke patients without dysphagia during the acute phase (83). Furthermore, stroke patients with dysphagia experienced larger reductions in AHI, particularly obstructive apneas, during recovery.

### Lesion Size

Few studies have examined how the size of stroke, regardless of whether the stroke in hemorrhagic or ischemic, effects SDB prevalence and type. Ahn et al. demonstrated that bilateral hemisphere lesions resulted in a significantly higher SDB severity compared to strokes that occurred in a single area (72). By contrast, Brown et al. compared brainstem to cortical strokes and showed that the lesion size was not associated with the severity of SDB (18). Siccoli et al. found that the worst SDB severity occurred with total anterior circulatory stroke, implying that the lesion size was a significant contributor to SDB severity (19). Yet, lesions occurring in the pons, a specific area of the brainstem, resulted in the second largest AHI, questioning whether the size of stroke is a contributing factor.

### Differences between Ischemic and Hemorrhagic Strokes on SDB Severity

Only a single study has examined SDB differences between ischemic and hemorrhagic strokes (78). SDB severity immediately post stroke was similar between ischemic and hemorrhagic strokes, but after 3 months, SDB severity remained unchanged in ischemic stroke, but was significantly reduced for those who experienced hemorrhagic stroke. The type of SDB was not recorded; thus, it is difficult to determine whether stroke type leads to differences in SDB type from this study. The authors suggest that the observed reduction in SDB in the hemorrhagic stroke group after 3 months may have been due to reductions in intracerebral pressure (73). More research is needed on the impact of stroke type on SDB as it appears that this information might be clinically meaningful when evaluating prognosis and therapy options in post-stroke SDB.

## Discussion

This review has highlighted that post-stroke SDB is significantly more prevalent compared to non-stroke populations. This is likely driven by lesion damage to respiratory control neurocircuitry in the brainstem and/or the cortex, and due to the complexity of these regions, the causes and the resulting type of SDB differ significantly between individual stroke patients. The literature on lesion location and size is limited and mixed with some studies reporting a link between brainstem stroke and SDB, in particular CSA, while others show cortical strokes to be more important and yet others showing no association between lesion location and SDB. It is too early to make strong conclusions on this, and further research is necessary to determine the link between stroke location/size and SDB.

Although SDB may reduce and resolve in severity in many patients over time, in parallel with functional recovery after stroke, SDB can significantly hinder and delay post-stroke rehabilitation, resulting in increased hospitalization and slower recovery. Importantly, the small number of studies that have examined the therapeutic effect of CPAP on recovery outcomes in stroke patients has reported significantly faster functional recovery, reduced hospitalization time, and frequency of re-hospitalization. This provides good rationale for attempting to treat post-stroke SDB to improve clinical outcomes, but clearly using CPAP in this patient group presents significant challenges due to disabling and varying impact of stroke, leading to limited CPAP adherence and use in hospital environment. There are several alternate options to treat SDB, such as MAS and supine avoidance that may be suitable in some patients to at least reduce SDB severity. New SDB-phenotyping techniques are being developed, which will inform regarding the nature and cause of SDB in individual patients, and as a result, new therapies are being developed to target phenotypic causes of SDB to provide tailored treatment for individual patients. Although at present many of these phenotyping techniques are complex and experimental, making them out of scope for clinical use, there are efforts to utilize simple sleep recordings from oximetry and flow measurements suitable for in-hospital assessment. These new phenotyping and therapy developments together with stroke location and size data could provide important clinical information to help prove SDB therapy in post-stroke and improve clinical outcomes for patients. In conclusion, the relative lack of studies examining SDB in the aftermath of stroke, along with the discrepancies in findings, highlights the need for more research in this area. Specifically, research links lesion location to SDB phenotypes toward informing what drives SDB post stroke in individual patients and guiding the choice of SDB treatment and stroke rehabilitation.

## Author Contributions

DS: manuscript concept, preparation, write-up, reviewing, and editing; RM: manuscript reviewing and editing; SM: manuscript reviewing and editing; AV: manuscript concept, preparation, write-up, reviewing, and editing.

## Conflict of Interest Statement

The authors declare that the research was conducted in the absence of any commercial or financial relationships that could be construed as a potential conflict of interest.

## Footnotes

^1^Patients in this study had focal lesions to the rostrolateral medulla; however, this study did not specify how the lesions occurred.
